# Simultaneous low-frequency vibration isolation and energy harvesting via attachable metamaterials

**DOI:** 10.1186/s40580-024-00445-2

**Published:** 2024-09-26

**Authors:** Jaeyub Hyun, Jaesoon Jung, Jeongwon Park, Wonjae Choi, Miso Kim

**Affiliations:** 1 Department of Mechanical Engineering, Pukyong National University, 45 Yongso-ro, Busan, 48513 Republic of Korea; 2 The 1st R&D Institute-9, Agency for Defense Development (ADD), P.O. Box 35, Yuseong-gu, Daejeon, 305-600 Republic of Korea; 3 Non-Destructive Metrology Group, Korea Research Institute of Standards and Science (KRISS), 267 Gajeong-ro, Yuseong-gu, Daejeon, 34113 Republic of Korea; 4 R&D Division, Advanced NDE Service Corporation (ANSCO), 419 Expo-ro, Yuseong-gu, Daejeon, 34051 Republic of Korea; 5 Department of Precision Measurement, University of Science and Technology (UST), 217 Gajeong-ro, Yuseong-gu, Daejeon, 34113 Republic of Korea; 6 School of Advanced Materials Science and Engineering, Sungkyunkwan University (SKKU), 2066 Seobu-ro, Jangan-gu, Suwon, 16419 Republic of Korea; 7 SKKU Institute of Energy Science and Technology (SIEST), Sungkyunkwan University (SKKU), 2066 Seobu-ro, Jangan-gu, Suwon, 16419 Republic of Korea

**Keywords:** Energy harvesting, Local resonance, Low-frequency vibration, Metamaterial, Piezoelectricity, Vibration suppression

## Abstract

In this study, we achieved energy localization and amplification of flexural vibrations by utilizing the defect mode of plate-attachable locally resonant metamaterials, thereby realizing compact and low-frequency vibration energy suppression and energy harvesting with enhanced output performance. We designed a cantilever-based metamaterial unit cell to induce local resonance inside a periodic supercell structure and form a bandgap within the targeted low-frequency range of 300–450 Hz. Subsequently, a defect area was created by removing some unit cells to break the periodicity inside the metamaterial, which led to the isolation and localization of the vibration energy. This localized vibration energy was simultaneously converted into electrical energy by a piezoelectric energy harvester coupled with a metamaterial inside the defect area. Consequently, a substantially enhanced energy harvesting output power was achieved at 360 Hz, which was 43-times higher than that of a bare plate without metamaterials. The proposed local resonant metamaterial offers a useful and multifunctional platform with the capability of vibration energy isolation and harvesting, while exhibiting easy handling via attachable designs that can be tailored in the low-frequency regime.

## Highlights

**•** Compact multifunctional vibration isolation-energy harvesting metastructures based on a low-frequency defect bandgap.
**•** Enhanced harvesting power by a factor of 43 over a wide frequency range of 310-380 Hz
**•** Easily mass-produced metamaterial unit cell via a laser-cutting process.
**•** Multifunctionality, i.e., vibration isolation and harvesting, proved by a carefully-designed experiment.

**Graphical Abstract:**

## Graphical abstract

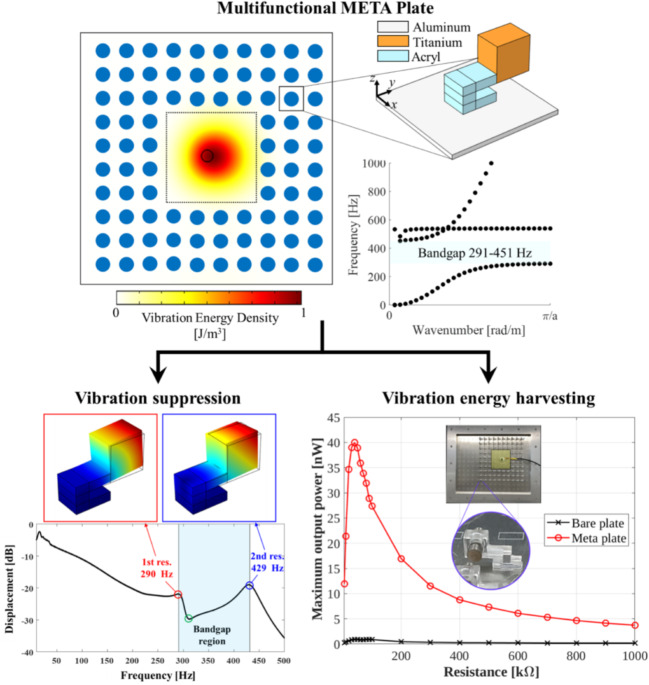

## Introduction

Metamaterials and phononic crystals (PCs) have emerged as a powerful platform for manipulating mechanical waves, such as acoustic, elastic, and vibrational waves in such a way that conventional materials cannot achieve. Exotic properties derived from their subwavelength scale and broad dynamic range allow for precise wave control, making them highly promising for a wide range of applications such as waveguides [1–3], wave protection [4], wave trapping [5, 6], and acoustic mirrors [7, 8]. Particularly pronounced is their potential in the low-frequency vibration regime, especially below 1 kHz, where they can markedly enhance performance in critical engineering applications like vibration isolation and energy harvesting (EH). For example, these materials can significantly reduce low-frequency vibrations in precision engineering machinery, where even minor disturbances can adversely impact operational performance (3–200 Hz) [9, 10]. Moreover, these materials are effective in mitigating low-frequency radiative noise in machinery and transportation systems, as most sound and noise are directly proportional to the structural vibration (50–200 Hz) [11, 12]. Beyond these applications, the extraordinary characteristics of metamaterials have also been investigated for their effectiveness in extremely low-frequency regimes, such as seismic wave protection in buildings (2–10 Hz) [13, 14], as well as in enhancing the performance of musical instruments (30–800 Hz) [15, 16]. Among these diverse applications, EH has gained significant attention due to its potential to convert ambient vibrations into usable electrical energy [17–20].

To harness the potential of metamaterials for EH, a variety of energy harvester types can be utilized,, including electromagnetic [21, 22], triboelectric [20, 23], electrostatic [24, 25], and piezoelectric energy harvesters [26–30]. Among these, the piezoelectric energy harvester (PEH) offers distinct advantages for use with industrial vibrating structures, primarily due to its ability to be directly applied to these structures. This direct integration facilitates the efficient conversion of mechanical vibrations into electrical energy, optimizing space utilization and simplifying the installation process. As a result, PEHs are particularly effective for low-frequency applications where compactness and efficiency are crucial. Therefore, our focus is on the PEH due to its ease of integration and high efficiency in converting mechanical energy into electrical energy. For a comprehensive review of piezoelectric energy harvesting technologies utilizing metamaterials across various applications, please refer to [17].

In the light of EH based on metamaterials, gradient-index (GRIN) PC structures play a crucial role, demonstrating significant potential when integrated with PEH [28, 31–34]. GRIN PC structures are designed to effectively refract waves by spatially modulating the effective refractive indices of the constituent unit cells, thereby enabling the focusing of the incident waves at a desired location. For instance, our recent studies have demonstrated the capability of an omnidirectional GRIN PC-based acoustic EH system to amplify acoustic waves from any direction towards a centrally located PEH [35]. This GRIN structure has also been successfully coupled with Helmholtz resonators and macrofiber composite-based PEH devices, leading to a significant enhancement in sound energy harvesting performance [36]. Furthermore, GRIN-based EH concepts have been explored not only in the audible acoustic regime but also in the ultrasonic elastic regime. In particular, these structures have been shown to optimize flexural energy harvesting performance through structural inverse design algorithms [30] and machine learning-enabled designs [37]. However, it is noteworthy that much of the existing research has concentrated on frequencies above 1 kHz. Challenges such as longer wavelengths and the necessity for compact designs limit the application of GRIN-based EH systems at frequencies below 1 kHz. Consequently, there is a notable gap in the research on the effective utilization of GRIN structures for low-frequency vibration energy harvesting.

On the other hand, locally resonant metamaterials (LRMs) are characterized by deep-subwavelength-scale microstructures that consist of locally resonating structural components. These structures enable effective manipulation of vibrational waves within the low-frequency regime [38–41]. Additionally, defect PC structures are known for their ability to create localized modes within a bandgap, where wave propagation is inhibited. By removing certain unit cells, wave energy can be concentrated and amplified within the specific region referred to as a defect [20, 42–49]. Given these distinctive features of the LRM and defect structures, they present promising candidates for low-frequency applications, including both vibration isolation or vibration EH.

The advent of metamaterials has led to significant advancements in achieving multi-functionality, such as simultaneous vibration isolation and EH. For example, a mechanical metamaterial has been designed for simultaneous mechanical wave filtering and EH through multiple PEHs embedded within a primary structural frame [38]. Similarly, a membrane-based acoustic metamaterial has been developed to provide both sound insulation and EH, although it does not address structural vibrations [50]. More recently, a dual-functional hierarchical mechanical metamaterial has been proposed for vibration isolation and energy absorption [51]. However, achieving the desired performance often necessitates substantial modifications to the underlying structures, which can be impractical for applications in industrial machinery. Additionally, the limited flexibility in tailoring these materials for specific target frequency ranges restricts their adaptability across various engineering contexts.

Therefore, in this study, we introduce a defect bandgap structure featuring two significant novelties: “*attachability*” and “*tailorability*”, designed specifically for simultaneous low-frequency vibration isolation and EH. The proposed attachable locally resonant metamaterial (a-LRM) unit cell can be easily integrated into existing structures without requiring alterations, providing greater flexibility and ease of use compared to the embedded metamaterials utilized in previous studies, which are often less versatile and more challenging to repurpose. Furthermore, the proposed defect bandgap structure offers tailorability, allowing performance customization through adjusting the geometric parameters or the mass of the cantilever tips within the a-LRM unit cell. This adaptability enhances its applicability across a range of frequency domains, making it an effective platform for low-frequency vibration isolation and EH. Additionally, introducing a defect into the a-LRM supercell can further isolate and localize vibrational energy within the defect region, enabling its conversion into electrical energy and thereby maximizing the EH performance.

## Methods/experimental

When the unit cells of an a-LRM are periodically arranged on a plate, a bandgap is created that blocks wave propagation. If a defect is created by intentionally removing LRMs, the mechanical wave energy is concentrated and amplified in the defect, resulting in a vibration mode commonly termed the defect mode. In this study, we refer to the plates with defect modes, such as a defect-bandgap structure. In this section, we first describe the modeling and analysis methods for the broadband response of flexural vibration energy localized within a defect of the defect bandgap structure as well as the bandgap features of the a-LRM unit cell. This is followed by a description of the experimental setup for characterizing vibration feature and energy harvesting performance of the a-LRM.

### Design of an attachable locally-resonant metamaterial (a-LRM) unit cell

**Fig. 1:**
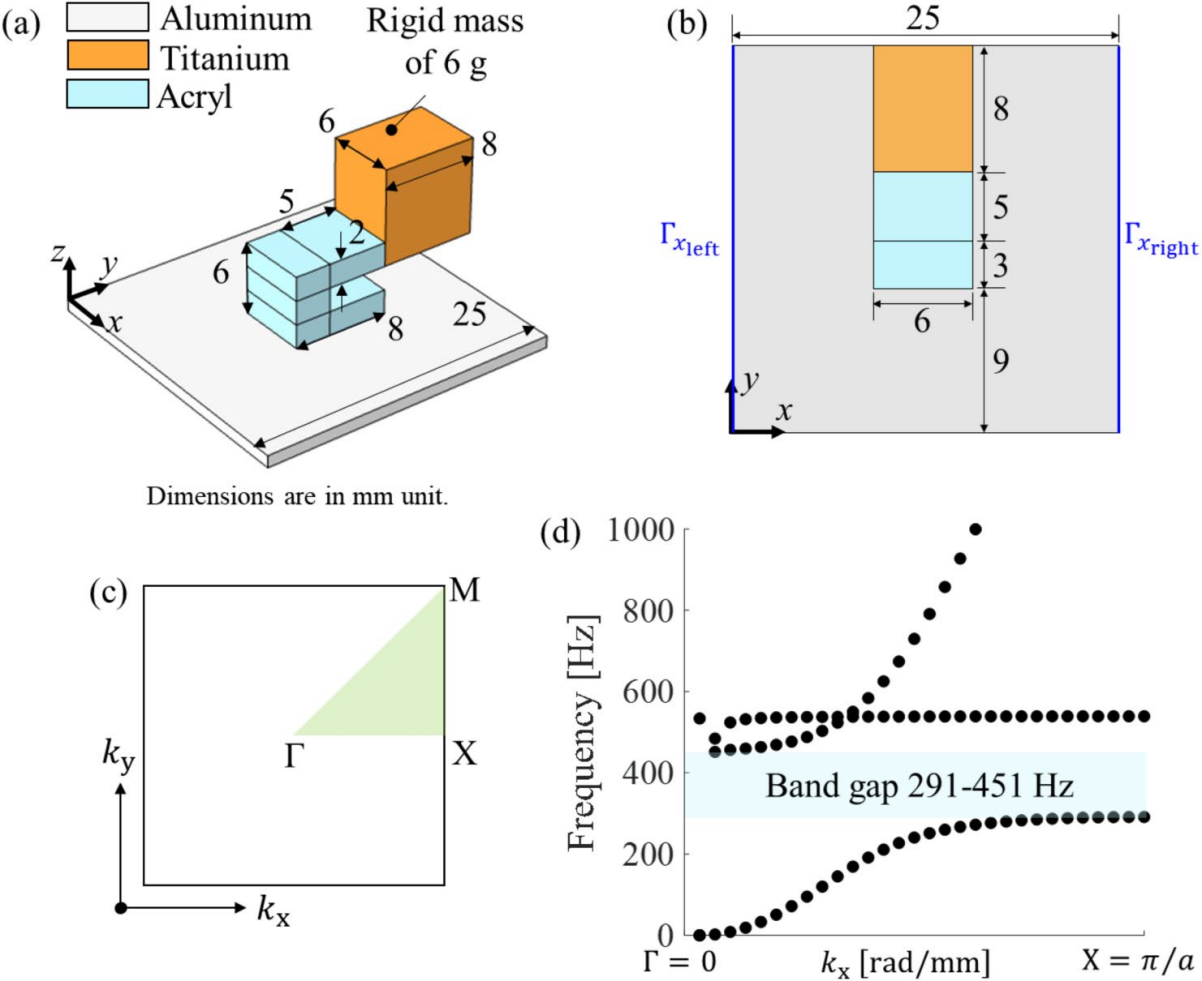
Dispersion analysis of the a-LRM unit cell and the local resonance-based band gap: (**a**) and (**b**) Isometric and top views of the designed a-LRM unit cell of a cantilever-type structure with a tip mass. (**c**) The irreducible first Brillouin zone represented by the green triangular area. (**d**) Dispersion curve (or band structure) of the designed a-LRM unit cell with a low-frequency bandgap in 291–451 Hz

In this study, as a building unit cell structure of an a-LRM for local resonance, we adopted a cantilever-type structure with a rigid mass at the tip (Fig. 1a); the structure is similar to the one proposed in a previous study where the structure was used for improving the sound radiation performance from a vibrational plate [52, 53]. This structure is easy to fabricate, and its operating frequency range can be tuned simply by varying the geometric parameters and tip mass properties. Acryl and titanium were used as the host beam and rigid tip mass, respectively. To design an a-LRM unit cell with a target low-frequency bandgap range of 300–450 Hz, we exploited the fact that the first and second bending resonance frequencies of the unit cell correspond to the start and end frequencies of the target bandgap range, respectively. The bending resonance frequencies of the a-LRM unit cell were experimentally verified to correspond to the start and end frequencies of the bandgap, as discussed in the next section.

Therefore, eigenfrequency analysis of the a-LRM unit cell was performed to calculate the bending resonance frequencies, and the dimensions and tip mass of the unit cell were adjusted to correspond to the target bandgap range. Because we used a titanium cylinder with a mass of 6 g as the tip mass for fabrication, the a-LRM unit cell was designed by adjusting only the dimensions of the unit cell while the tip mass was fixed at 6 g for practical convenience. The final dimensions are shown in Fig. 1a and b. It is worthy to note that a systematic structural optimization such as topology optimization can be utilized to match the bending-mode-induced bandgap to the desired low-frequency range specified [54, 55]. Although this optimization falls outside the scope of the current study and will be addressed in future research, we will explore the effects of the geometric parameters of the a-LRM unit cell on bending resonance frequencies through a parametric study in Sect. 3.1.

### Dispersion analysis of the designed a-LRM unit cell

The designed a-LRM unit cell is modeled using a full 3D solid model, and the partial differential governing equation of the time-harmonic wave motion for the linear elastic structure of an isotropic material without any body or traction forces is expressed as Eq. (1).

1 $$\:\nabla\:\cdot\:\varvec{\sigma\:}+\rho\:{\omega\:}^{2}\mathbf{u}=0,$$

where $\:\varvec{\sigma\:}$ is the Cauchy stress tensor, $\:\rho\:$ is the mass density, $\:\omega\:$ is the angular frequency, and $\:\mathbf{u}={\left[u\:v\:w\right]}^{\text{T}}$ is the displacement vector for Cartesian coordinates. To further detail this equation, $\:\varvec{\sigma\:}$ is expressed by $\:\varvec{\sigma\:}=\lambda\:\text{t}\text{r}\left(\varvec{\epsilon\:}\right)\mathbf{I}+2\mu\:\varvec{\epsilon\:}$, where $\:\varvec{\epsilon\:}=0.5\left(\nabla\:\mathbf{u}+\mathbf{u}\nabla\:\right)$ is the strain tensor, while $\:\lambda\:$ and $\:\mu\:$ are the Lamé constants. By applying the standard Galerkin approach to Eq. (1), the finite element (FE) governing equation for wave motion can be expressed in matrix notation, as shown in Eq. (2), where$\:\:\mathbf{K}\:$is the assembled stiffness matrix,$\:\:\mathbf{M}\:$is the assembled mass matrix, and $\:\mathbf{U}\:$is the nodal displacement vector.

2 $$\:\left(\mathbf{K}-{\omega\:}^{2}\mathbf{M}\right)\mathbf{U}=0$$

For the dispersion analysis of a periodic a-LRM unit cell, the wave motion was characterized using the Floquet–Bloch theorem, which defines the phase relationship of the displacements between the exterior boundaries of a unit cell, as expressed by Eq. (3).

3 $$\:\mathbf{U}\left(\mathbf{r}+\mathbf{a}\right)=\mathbf{U}\left(\mathbf{r}\right){e}^{j\mathbf{k}\cdot\:\mathbf{a}},$$

where $\:\:\mathbf{r}={\left[x\:y\:z\right]}^{\text{T}}$ is the position vector, $\:\:\mathbf{a}\:$ is the spatial periodicity (that is, the unit cell size, $\:a=25\:\text{m}\text{m}$), and $\:\:\mathbf{k}={\left[{k}_{x}\:{k}_{y}\:{k}_{z}\right]}^{\text{T}}\:$is the Bloch wave vector for the irreducible Brillouin zone, represented by the green triangular area in Fig. 1c.

To characterize the dispersion behavior of the a-LRM unit cell, it is sufficient to perform dispersion analysis only for the Bloch wave vectors in either the *x*- or *y*-directions. This is because the main mechanism responsible for forming the bandgap of the a-LRM is local resonance based on the flexural vibration mode and not Bragg scattering based on periodicity. Thus, we perform the dispersion analysis only for the *x*-direction in the range of $\:{k}_{x}$ from $\:0$ to $\:\pi\:/a$.

Based on this phase relationship (Eq. (3)), the condensed set ($\:\widehat{\mathbf{U}}$ with a size of $\:m$) is related to the full set ($\:\widehat{\mathbf{U}}$ with a size of $\:n$) of nodal displacements according to $$\:\mathbf{U}=\mathbf{P}\widehat{\mathbf{U}}=\mathbf{P}{\left[\begin{array}{ccc}{\mathbf{U}}_{\text{i}\text{n}\text{t}\text{e}\text{r}\text{n}\text{a}\text{l}}&\:{\mathbf{U}}_{{x}_{\text{l}\text{e}\text{f}\text{t}}}&\:{\mathbf{U}}_{{x}_{\text{r}\text{i}\text{g}\text{h}\text{t}}}\end{array}\right]}^{\text{T}}$$, where $\:\:\mathbf{P}\:$ is the $\:n\:\times\:\:m$ transformation matrix enforcing the periodic boundary condition. For a square unit cell with *x*-periodicity, $\:\mathbf{P}\:$has the following block form:

4 $$\:\mathbf{P}={\left[\begin{array}{ccc}{\mathbf{I}}_{\text{i}\text{n}\text{t}\text{e}\text{r}\text{n}\text{a}\text{l}}&\:0&\:0\\\:0&\:{\mathbf{I}}_{{x}_{\text{l}\text{e}\text{f}\text{t}}}&\:{\mathbf{I}}_{{x}_{\text{l}\text{e}\text{f}\text{t}}}{e}^{j{k}_{x}a}\end{array}\right]}^{\text{T}},$$

where $\:\:{\mathbf{I}}_{\text{i}\text{n}\text{t}\text{e}\text{r}\text{n}\text{a}\text{l}}$ and $\:{\mathbf{I}}_{{x}_{\text{l}\text{e}\text{f}\text{t}}}$ are the identity matrices corresponding to nodal displacement vectors $\:{\mathbf{U}}_{\text{i}\text{n}\text{t}\text{e}\text{r}\text{n}\text{a}\text{l}}$ and $\:{\mathbf{U}}_{{x}_{\text{l}\text{e}\text{f}\text{t}}}$, respectively. Then, applying Eq. (4) to Eq. (2) and employing the Galerkin projection, the FE equation considering periodicity, which is used for dispersion analysis, is obtained as follows:

5 $$\:\left(\widehat{\mathbf{K}}\left(\mathbf{k}\right)-{\omega\:}^{2}\widehat{\mathbf{M}}\left(\mathbf{k}\right)\right)\widehat{\mathbf{U}}=0,$$

where $\:\widehat{\mathbf{K}}={\mathbf{P}}^{\text{H}}\mathbf{K}\mathbf{P}$ and $\:\widehat{\mathbf{M}}={\mathbf{P}}^{\text{H}}\mathbf{M}\mathbf{P}$ denote the condensed stiffness and mass matrices, respectively. Here, $\:{(\bullet\:)}^{\text{H}}$ is the conjugate transpose operator. For detailed expressions of these matrices, please refer to [56]. To perform a dispersion analysis of the a-LRM unit cell, we used the built-in structural mechanics module with the full 3D solid model in COMSOL Multiphysics, a commercial FE analysis software package. To be more specific about the dispersion analysis using COMSOL Multiphysics, we first extract the $\:\mathbf{K}$ and $\:\mathbf{M}$, the assembled stiffness and mass matrices in Eq. (2) with the LiveLink for MATLAB which is the MATLAB interface of COMSOL Multiphysics. Then, with the degree-of-freedom information of FE model we construct the transformation matrix $\:\mathbf{P}$ for the periodic boundary conditions given in Eq. (4), and finally obtain Eq. (5) through the Galerkin projection, i.e., $\:\widehat{\mathbf{K}}={\mathbf{P}}^{\text{H}}\mathbf{K}\mathbf{P}$ and $\:\widehat{\mathbf{M}}={\mathbf{P}}^{\text{H}}\mathbf{M}\mathbf{P}$ on the previously extracted $\:\mathbf{K}$ and $\:\mathbf{M}$. We then perform the eigenvalue analysis of Eq. (5) by looping through the wavenumber $\:{k}_{x}$ to obtain the dispersion curve, i.e., the $\:{k}_{x}\text{-}f$ curve, with $\:f$ being the frequency. The properties of the materials used (aluminum, acrylic, and titanium) are listed in Table 1. Figure 1d shows the dispersion curve (or band structure) calculated via dispersion analysis, where a bandgap is formed in the frequency range of 291–451 Hz, which is very close to the targeted low-frequency regime.

**Table 1:** Properties of materials used (nominal values at 1 atm and 20 ℃) [57]. The Lamé constants of homogeneous isotropic linear elastic materials are determined as $\:\lambda\:=\frac{E\nu\:}{\left(1+\nu\:\right)\left(1-2\nu\:\right)}$ and $\:\mu\:=\frac{E}{2\left(1+\nu\:\right)}$

	Young’s modulus $(\:E,\:\text{G}\text{P}\text{a})$	Poisson’s ratio $(\:\nu\:)$	Mass density $(\:\rho\:,\:\text{k}\text{g}/{\text{m}}^{3})$	Lamé first constant $(\:\lambda\:,\:\text{P}\text{a})$	Lamé second constant $(\:\mu\:,\:\text{P}\text{a})$
Aluminum	65.0	0.30	2,700	37.5	25
Acryl	3.1	0.38	1,165	3.6	1.1
Titanium	120.0	0.34	4,500	95.1	44.8

### Experimental setup for vibration characteristic of the a-LRM unit cell

First, to manufacture the a-LRM unit cell, the host beam was fabricated using the laser-cutting process of a transparent acrylic sheet with a thickness of 6 mm, and a commercially available titanium cylinder (6 g) was used as a rigid mass. Then, the a-LRM unit cell was manufactured by assembling these two parts, and each unit cell was attached to an aluminum plate of 1 mm thickness using silicon glue. This a-LRM unit cell can be easily tuned to achieve a specific bandgap range by adjusting the geometric and material properties of the rigid mass and cantilever.

**Fig. 2:**
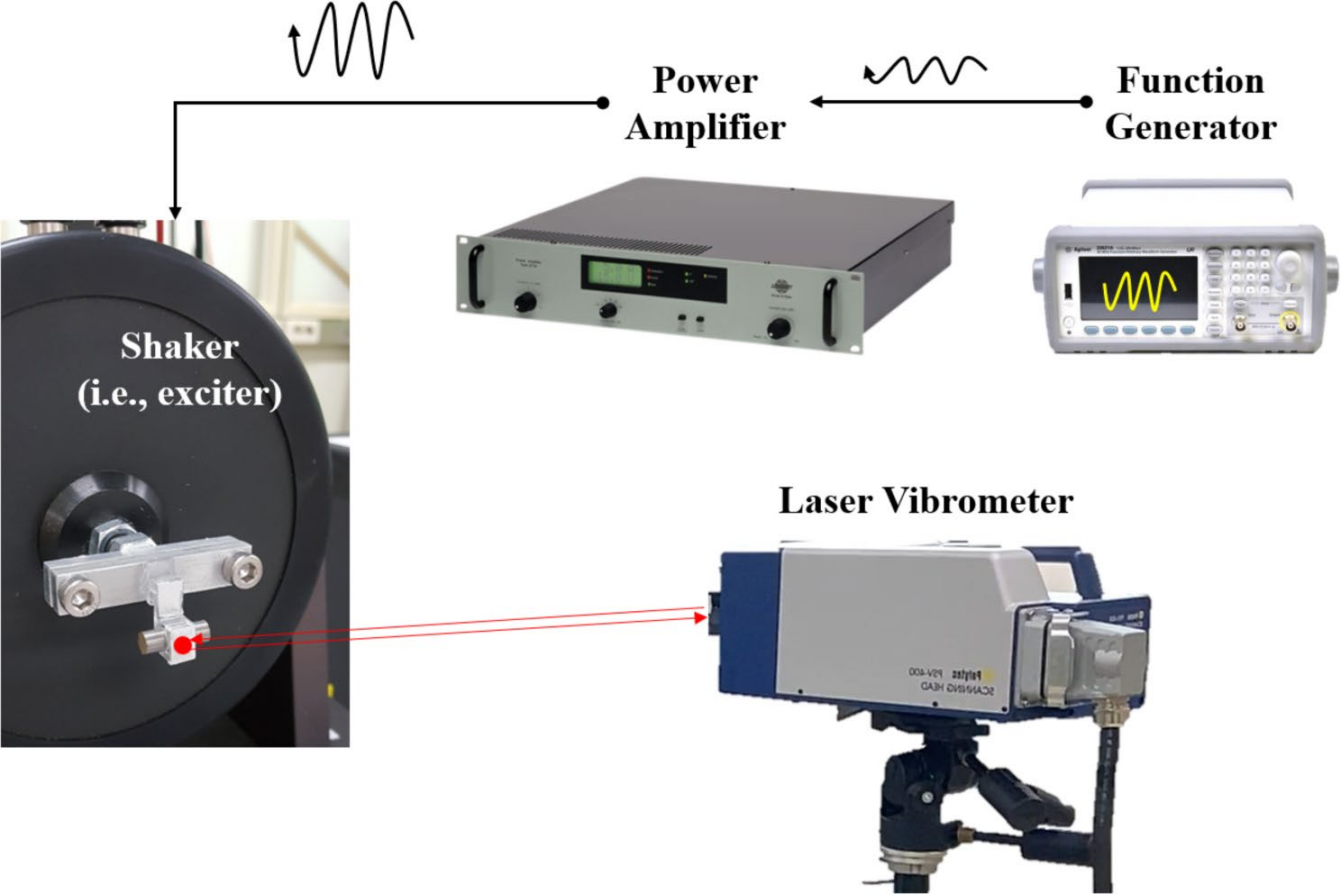
Experimental setup for measuring the vibration characteristic of the a-LRM unit cell

The a-LRM was designed based on the fact that the first and second bending resonance frequencies of the unit cell correspond to the start and end frequencies of the target bandgap range, respectively. Therefore, we show experimentally that this is sufficiently valid for our a-LRM design. The configuration of the experimental setup used to evaluate the vibration characteristics of the designed a-LRM unit cell is shown in Fig. 2. The actuation unit comprised a function generator (Agilent, 35500 B Series), power amplifier (Bruel & Kjaer, Type 2718), and vibration shaker (Bruel & Kjaer, Type 4809). The a-LRM unit cell was attached to a base beam with a larger mass using silicon glue to measure the flexural vibration response of the a-LRM unit cell as independently as possible from that of the host structure (that is, the base beam). A sweep function from 10 to 500 Hz was applied to excite the base beam with the a-LRM unit cell for 10 s using a shaker, and the time response of the mechanical displacement at the center of mass (that is, the tip of the cantilever) was measured using a laser vibrometer (Polytec, OFV-505, OFV-5000).

**Fig. 3:**
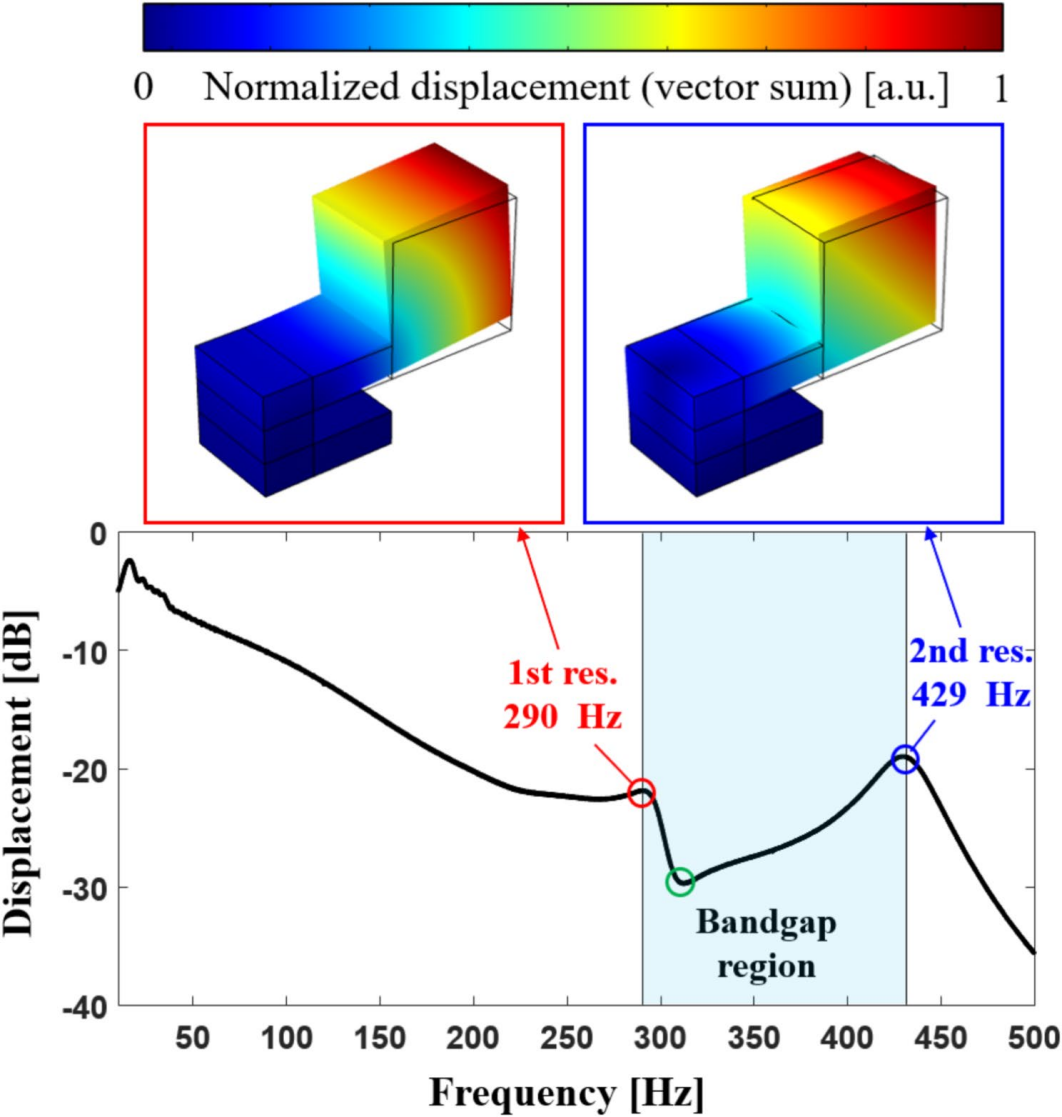
Experimentally measured frequency response of displacement at the center of mass of the a-LRM unit cell: red and blue circles denote the first and second bending resonance frequencies, respectively, and their corresponding numerically calculated eigenmodes are represented in the upper boxes. The green circle indicates the anti-resonance frequency where the displacement is minimized

The frequency response of the measured displacement at the center of mass of the a-LRM unit cell, as shown in Fig. 3, was obtained by applying a fast Fourier transform to the time response of the displacement to identify the resonant modes. Notably, the first and second bending resonance frequencies, represented by red and blue circles, correspond to the start and end frequencies of the bandgap region, respectively. The corresponding numerically calculated eigenmodes are indicated in the upper box. The green circle indicates that the dips in the frequency and displacement are very small. This dip physically exhibited an anti-resonance frequency, manifested through the combination of the first and second bending resonance modes.

### Experimental setup for flexural vibration amplification and EH via the a-LRM

**Fig. 4:**
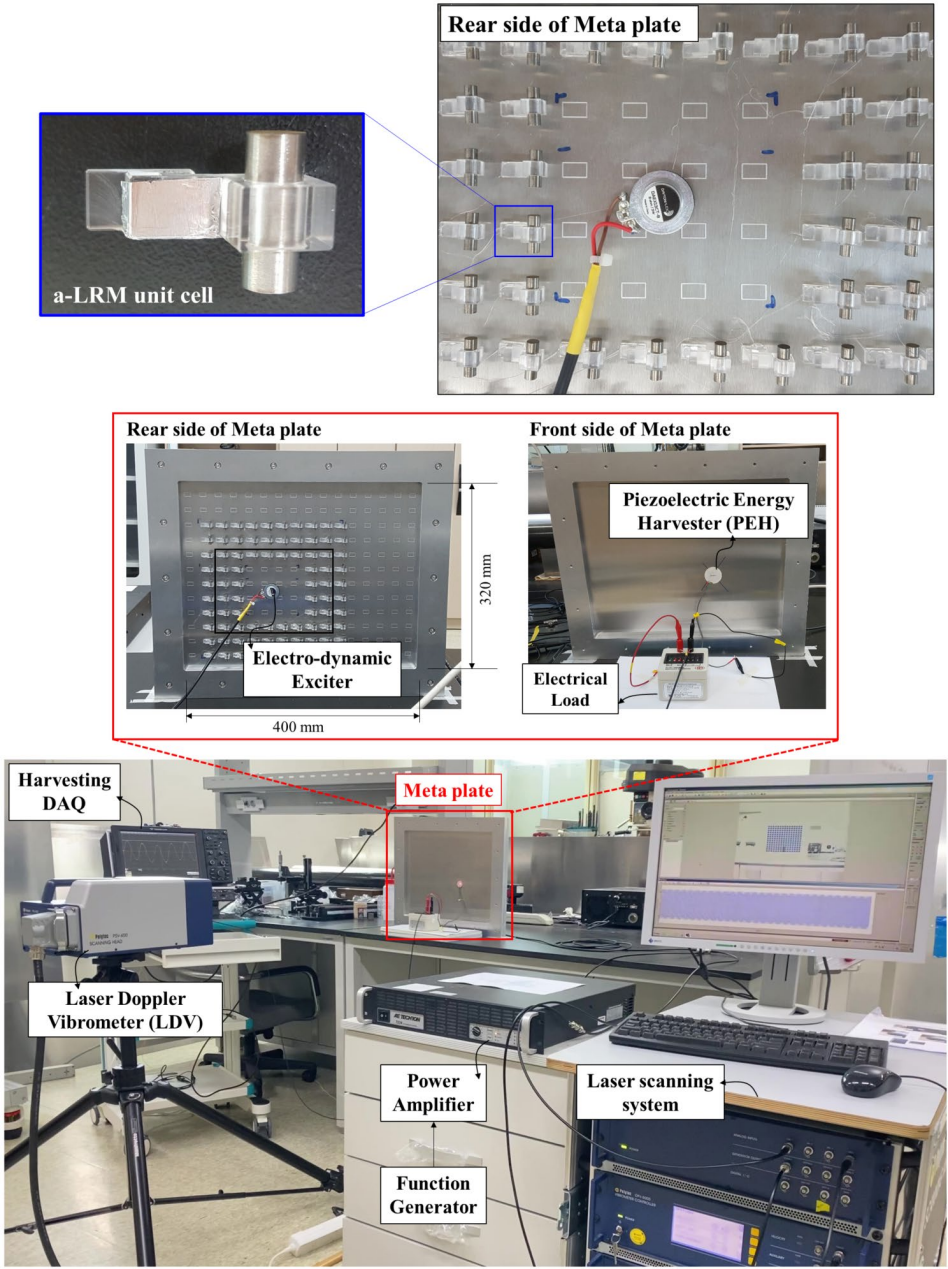
Overall experimental setup for both the vibration localization/amplification and EH performance characterization: photographs of the overall experimental setup comprising excitation and energy-harvesting parts

To experimentally evaluate and characterize both the flexural vibration localization and amplification performance as well as the EH capability, two types of aluminum plates with and without a-LRMs were employed, where the first is termed the *meta-plate* and the latter is termed the *bare plate*; the bare plate has the same material and geometric properties as that of the meta-plate. We used a $\:400\times\:320\:\text{m}\text{m}$ aluminum bare plate with a thickness of 1 mm as the reference system; the experimental setup is shown in Fig. 4. The input wave generation unit comprised a function generator (AFG3051C, Tektronix), a power amplifier (7224, AE Techron), and an electrodynamic exciter (DAEX13CT-8, Dayton Audio). This electrodynamic exciter should be placed at the center of the defect. A PEH and a piezoelectric ceramic disc $$\begin{aligned}(23\text{P}\text{b}\text{Z}\text{r}{\text{O}}_{3}-36\text{P}\text{b}\text{T}\text{i}{\text{O}}_{3}&-41\text{P}\text{b}\left(\text{N}\text{i}1/3\text{N}\text{b}2/3\right){\text{O}}_{3}\\&{\text{, 3PZ-36PT-41PNN}})\end{aligned}$$ with a diameter of 24 mm and a thickness of 2 mm with electrodes were attached at the center position of the defect for EH. The PEH was connected to an electrical load so that the voltage and power across various electrical loading conditions could be measured. For stable measurements, the power amplifier was turned on for at least 30 min before the start of the measurement.

## Results and discussion

### Effect of the geometrical parameters of the a-LRM unit cell on bending frequency

**Fig. 5:**
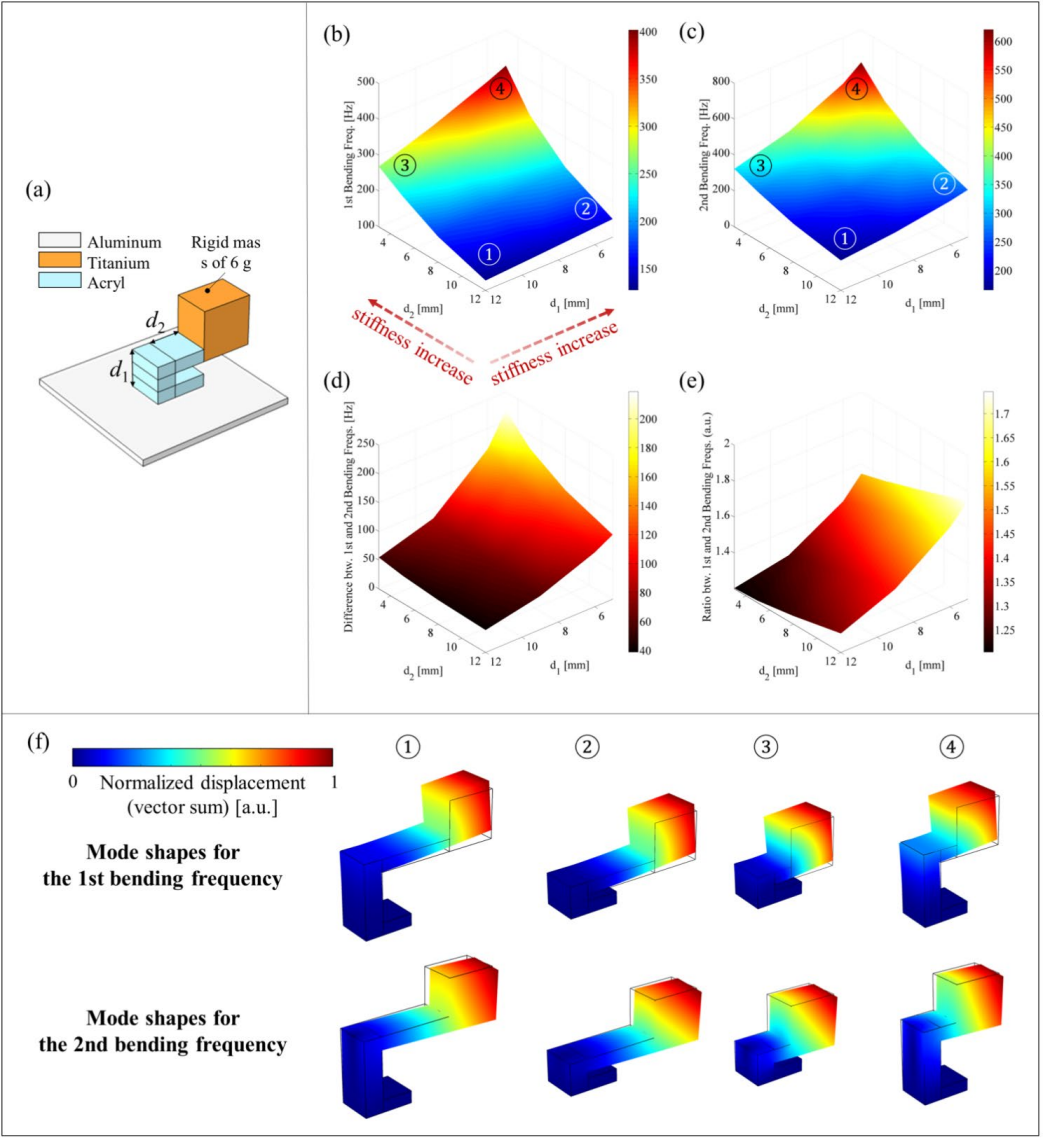
Effect of the two design parameters $\:{d}_{1}$ and $\:{d}_{2}$ on the bending resonance frequency of the a-LRM unit cell: (**a**) The a-LRM unit cell with the two design parameters. (**b**) The first resonance frequency. (**c**) The second resonance frequency. (**d**) Difference between the first and second resonance frequencies. (**e**) Ratio of the second to first resonance frequencies. (**f**) Normal mode shapes for the first resonance frequency (upper row) and the second one (bottom row), and the normal mode is represented by vector summation of displacement, i.e., $\:||\mathbf{u}||=\sqrt{{\text{u}}^{2}+{\text{v}}^{2}+{\text{w}}^{2}}$

The bending resonance frequency is conceptually defined as $\:\sqrt{{k}_{b}/m}$ where $\:{k}_{b}$ is the bending stiffness and $\:m$ is the mass. This relationship indicates that the bending resonance frequency is directly proportional to the bending stiffness and inversely proportional to the mass. Specifically, the first bending resonance frequency of a cantilever beam, a key structural component of the a-LRM unit cell, is influenced by its geometric dimensions, including beam length and bending section thickness. The mass varies linearly with these dimensions, while the stiffness varies nonlinearly due to the area moment of inertia (also known as the second moment of area), which implies that the stiffness has a more significant impact on the bending resonance frequency. Therefore, in order to effectively change the bending stiffness of the a-LRM unit cell, a parametric study is conducted by selecting two design parameters $\:{d}_{1}$ and $\:{d}_{2}$ corresponding to the length and thickness of the beam as shown in Fig. 5a.

The bending stiffness increases as both design parameters $\:{d}_{1}$ and $\:{d}_{2}$ decrease, thereby increasing both the first and second bending resonance frequencies, as illustrated in Fig. 5b and c. The corresponding values for the first and second resonance frequencies are presented in Tables 2 and 3, respectively.

**Table 2:** First bending resonance frequencies of the a-LRM unit cell according to the two design variables ${d}_{1}$ and $\:{d}_{2}$

First bending resonance frequency (Hz)		$\bf{d}_{1}$ (mm)			
		5	6	9	12
$\:{d}_{2}$ (mm)	3	401.6	378.9	319.1	268.3
	5	303.2	295.2	254.4	224.8
	8	216.4	209.8	188.1	171.2
	12	150.4	146.9	136.7	127.3

**Table 3:** Second bending resonance frequencies of the a-LRM unit cell according to the two design variables $\bf{d}_{1}$ and $\:{d}_{2}$

Second bending resonance frequency (Hz)		$\bf{d}_{1}$ (mm)			
		5	6	9	12
$\:{d}_{2}$ (mm)	3	620.2	543.5	402.2	322.9
	5	480.1	432.1	329.1	272.3
	8	357.1	324.7	254.3	213.3
	12	262.6	241.1	194.9	166.1

It is particularly evident that the design parameter $\:{d}_{2},$ which corresponds to the beam length has a greater influence on the change in the bending resonance frequency than $\:{d}_{1}$. This effect is especially pronounced for the first bending resonance frequency. Figure 5d illustrates the relationship between the first and second resonance frequencies, demonstrating that as the bending stiffness increases, the frequency difference also increases. Interestingly, $\:{d}_{1}$ has a more substantial impact on the difference between the resonant frequencies than $\:{d}_{2}$. Since the difference in resonant frequencies is directly related to the width of the bandgap, this suggests that to achieve a wider bandgap, adjusting $\:{d}_{1}$ is more effective than modifying $\:{d}_{2}$.

On the other hand, for effective vibration applications, not only the width of bandgap but also its center frequency is an important design factor, highlighting the necessity to search the proper $\:{d}_{1}$ and $\:{d}_{2}$. This can be supported by the ratio of the second to first resonance frequencies, i.e., $\:{f}_{2}/{f}_{1}$, where $\:{f}_{2}$ and $\:{f}_{1}$ represent the second and first resonance frequencies, respectively, shown in Fig. 5e. To this end, either optimization methods [58] or artificial intelligence [59] can be adopted; however, the exploration of these approaches is reserved for future work.

### Frequency response of flexural vibration of the defect bandgap structure

**Fig. 6:**
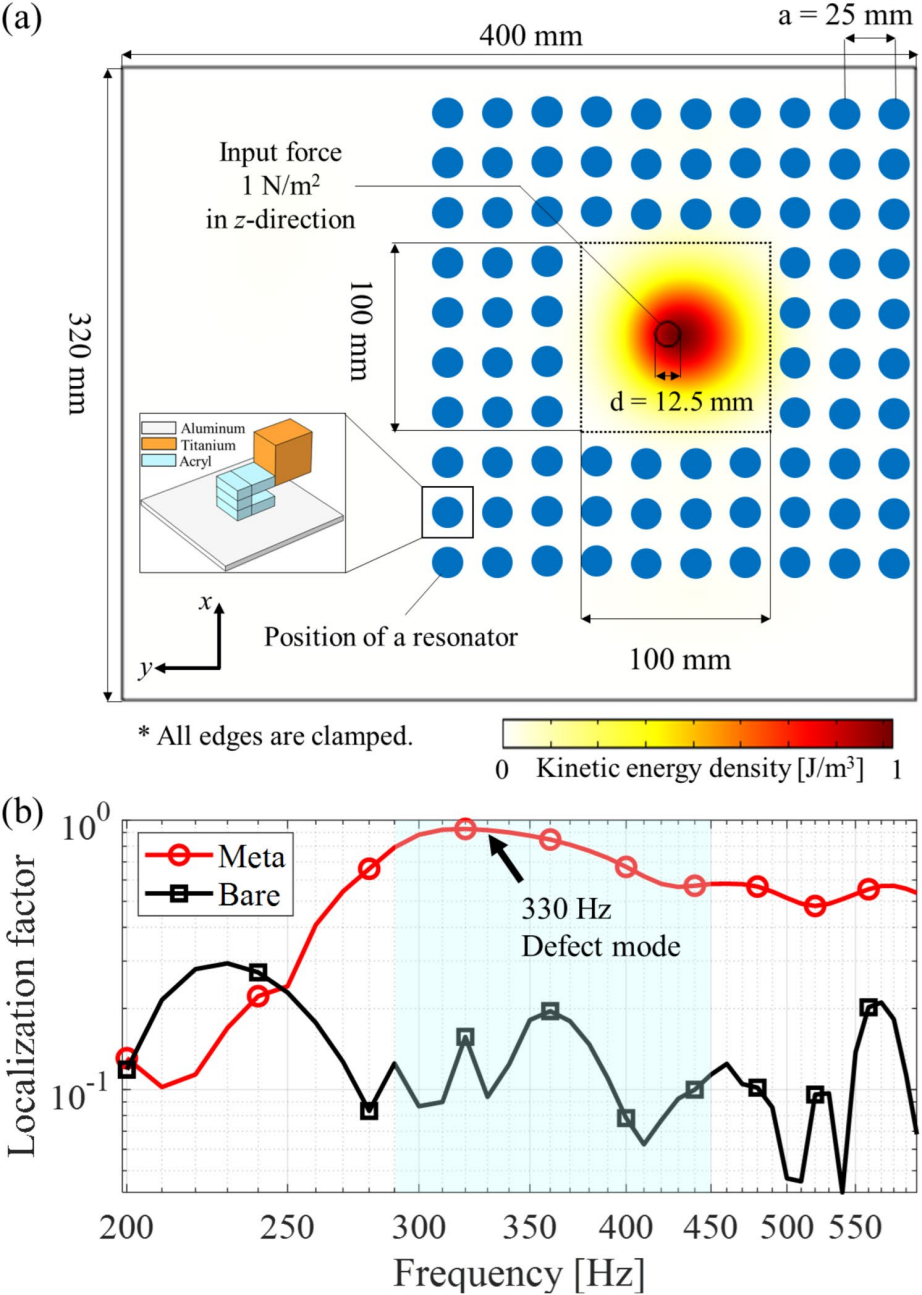
(**a**) Defect bandgap structure with the corresponding kinetic energy density normalized to the maximum value. Here, each resonator position corresponds to the a-LRM unit cell. The inset shows the designed a-LRM unit cell of the cantilever-type structure with a tip mass for local resonance. (**b**) Calculated frequency response of kinetic energy localization factor for the a-LRM and bare aluminum plate

Figure 6a illustrates a plane view of the proposed a-LRM design, where a supercell comprising 10 × 10 local resonant unit cells is attached to a thin aluminum plate. Time-harmonic analysis was performed using COMSOL Multiphysics by sweeping the frequency range from 200 to 600 Hz with a step frequency of 10 Hz to calculate the frequency response of the flexural vibration energy localized within the defects of the bandgap structure. Here, the a-LRM unit cells of $\:10\times\:10$ arrangements were modeled, assuming perfect bonding to a 1-mm aluminum base plate. The locations of the a-LRM unit cells are represented by blue circles in Fig. 6a. Clamped boundary conditions were imposed on the exterior boundaries of the aluminum base plate to model the perfect reflection of the elastic waves. The maximum allowable mesh size was set to 1/10 of the wavelength to obtain sufficient spatial resolution for wave propagation. An input force of 1 $\:\text{N}/{\text{m}}^{2}$ in the *z*-direction was applied at the center of the defect to excite only the flexural wave. Since the designed flexural EH system is based on a-LRM targets, where the input wave position is specified, an excitation is generated within the defect to effectively confine and amplify the mechanical energy. Additionally, in the case of the bare plate without a-LRMs, effective excitation could not be achieved when the input excitation position was placed on a nodal line at which the amplitude of the flexural vibration was zero. Therefore, the input position was shifted in the + *x* direction from the center of the defect bandgap structure to minimize the possibility of nodal line issues. The flexural energy is well localized and amplified within the defect, as shown in Fig. 6a. Interestingly, the peak of the flexural defect mode localized within the defect occurs approximately 12.5 mm away from the input excitation position, because the periodic arrangement of the a-LRM unit cells was located close to the top-right boundaries of the defect bandgap structure.

To quantify the degree of flexural energy localization, we introduce a localization factor, which is defined as the ratio of the integral of the kinetic energy of the defect to that of the overall aluminum base plate. Here, the localization factor is expressed by the flexural displacement $\:w$ only, as our main target is out-of-plane flexural waves.

7 $$\:\text{L}\text{o}\text{c}\text{a}\text{l}\text{i}\text{z}\text{a}\text{t}\text{i}\text{o}\text{n}\:\text{f}\text{a}\text{c}\text{t}\text{o}\text{r}=\frac{{\int\:}_{{{\Omega\:}}_{\text{d}\text{e}\text{f}\text{e}\text{c}\text{t}}}^{}\rho\:w{w}^{*}\text{d}{\Omega\:}}{{\int\:}_{{{\Omega\:}}_{\text{o}\text{v}\text{e}\text{r}\text{a}\text{l}\text{l}}}^{}\rho\:w{w}^{*}\text{d}{\Omega\:}},$$

where $\:{(\bullet\:)}^{\varvec{*}}$ denotes a complex conjugate operator. The calculated localization factor response is shown in Fig. 6b. Compared with the bare plate without a-LRMs (bare), the defect bandgap structure (meta-plate) shows a larger localization factor response in the frequency range of approximately 250–600 Hz. Additionally, the localization factor at the defect-mode frequency of 330 Hz was close to one, indicating that almost all the flexural kinetic energy was localized in the defect.

The localization factor serves as an effective metric for evaluating the overall localization performance of the defect bandgap structure (meta-plate); however, it is not suitable for characterizing the defect itself, specifically the cavity. To independently evaluate the performance of the cavity alone, the Purcell factor can be utilized, which is numerically calculated as the ratio between the power densities of the bandgap structures with and without defects [60–62].

**Fig. 7:**
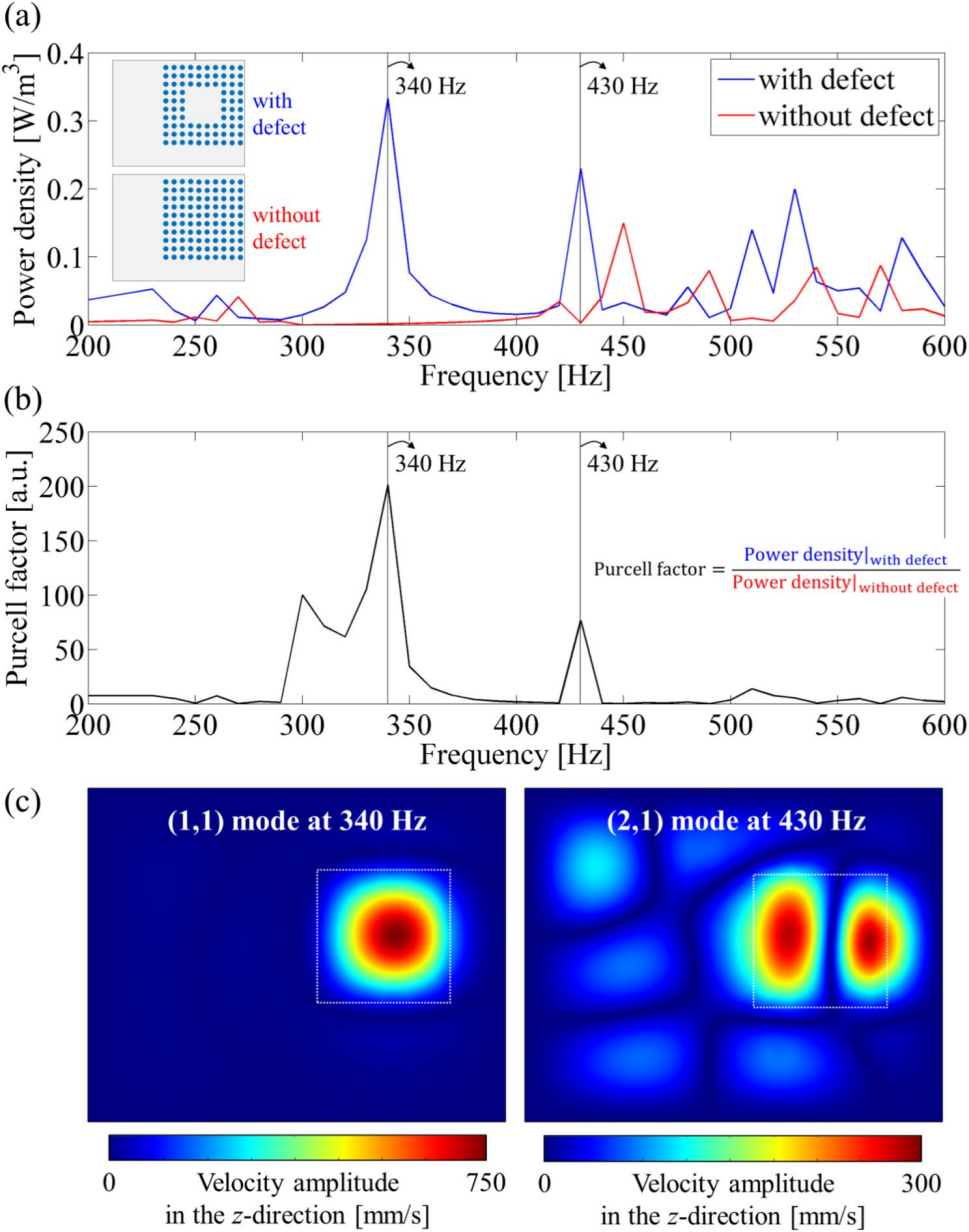
Frequency response of the Purcell factor numerically calculated: (**a**) Power density responses for the bandgap structure with and without defects. Here, the power density is calculated only for the defect region. (**b**) Purcell factor response. (**c**) Velocity amplitude fields corresponding to the two defect modes, i.e., (1,1) and (2,1) modes at 340 Hz and 430 Hz

As shown in Fig. 7a and b, we performed frequency response analysis over the frequency range of 200 to 600 Hz and calculated the Purcell factor response. Here, power densities were calculated exclusively for the defect regions. Notably, the Purcell factor response in Fig. 7b reveals that two main peaks occur at 340 Hz and 430 Hz. Furthermore, as shown in Fig. 7c, vibration energy is concentrated within the defect only at these frequencies. Indeed, these peaks correspond to the (1,1) and (2,1) defect modes, respectively, which will be analyzed in more detail in the following.

**Fig. 8:**
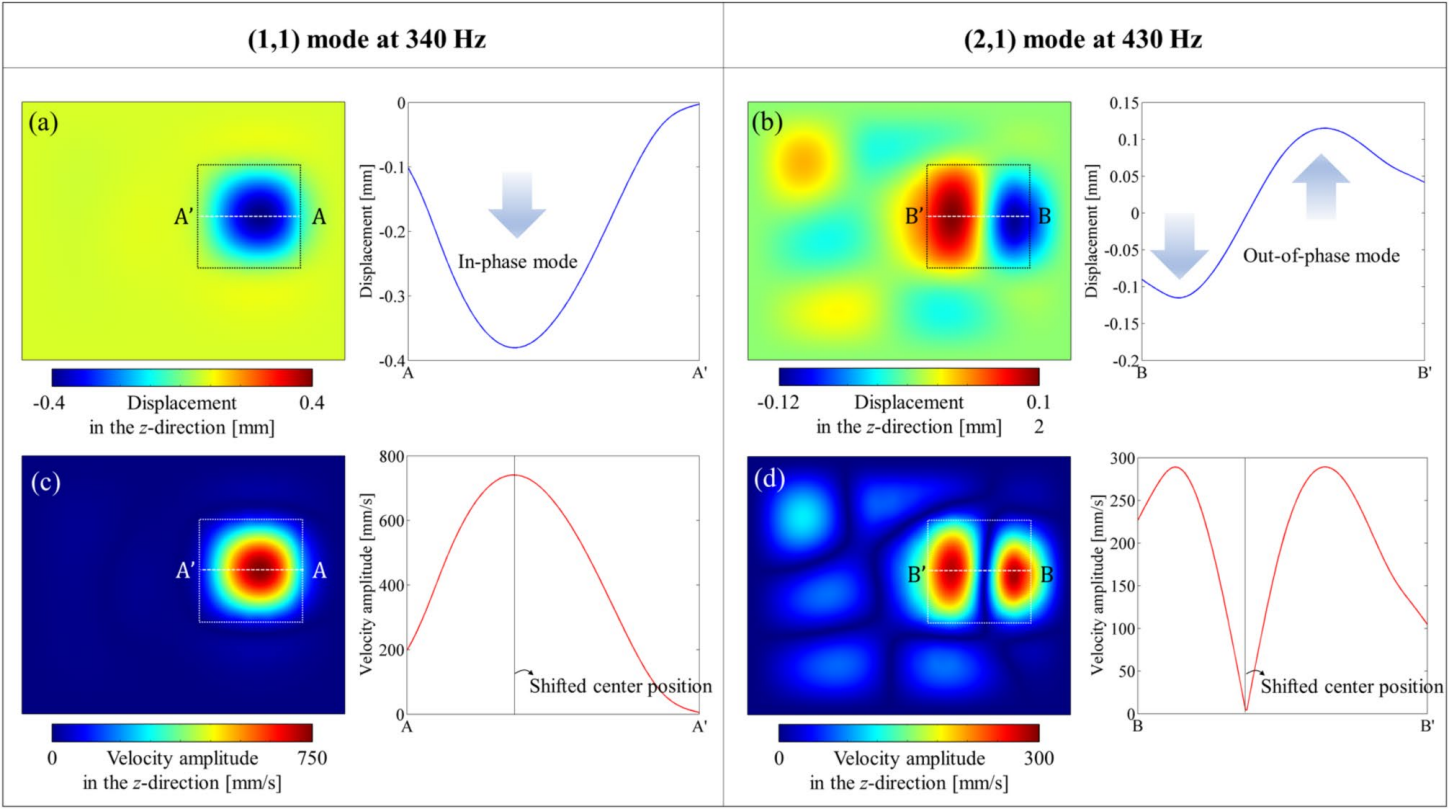
The vibration fields numerically calculated for the two mode frequencies: (**a**) and (**c**) Displacement and velocity amplitude fields for the (1,1) mode at 340 Hz. (**b**) and (**d**) Displacement and velocity amplitude fields for the (2,1) mode at 430 Hz

The frequency response of the Purcell factor presented in Fig. 7 identifies two frequencies, 340 Hz and 430 Hz, as the (1,1) and (2,1) defect modes, respectively. Figure 8 displays the vibrational fields calculated for these two frequencies, effectively illustrating the occurrence of both defect modes with amplified vibration energy density. Notably, the centers of these modes are slightly displaced to the right of the defect center, which can be attributed to the defect bandgap structure being attached to an aluminum plate with a rightward bias.

As shown in Fig. 8a and c, the (1,1) defect mode at 340 Hz exhibits a large velocity amplitude and an in-phase behavior. In contrast, the (2,1) defect mode at 430 Hz demonstrates out-of-phase behavior, which is expected to reduce energy conversion efficiency due to voltage cancellation throughout the PEH, as illustrated in Fig. 8b and d. This voltage cancellation effect is well-documented and arises from the sign change of mechanical strain associated with the out-of-phase mode [29, 63]. Consequently, although higher-order modes, including the (2,1) mode, are noted, the (1,1) defect mode is selected as the primary target for evaluating PEH performance.

### Effect of the defect size on flexural defect modes

**Fig. 9:**
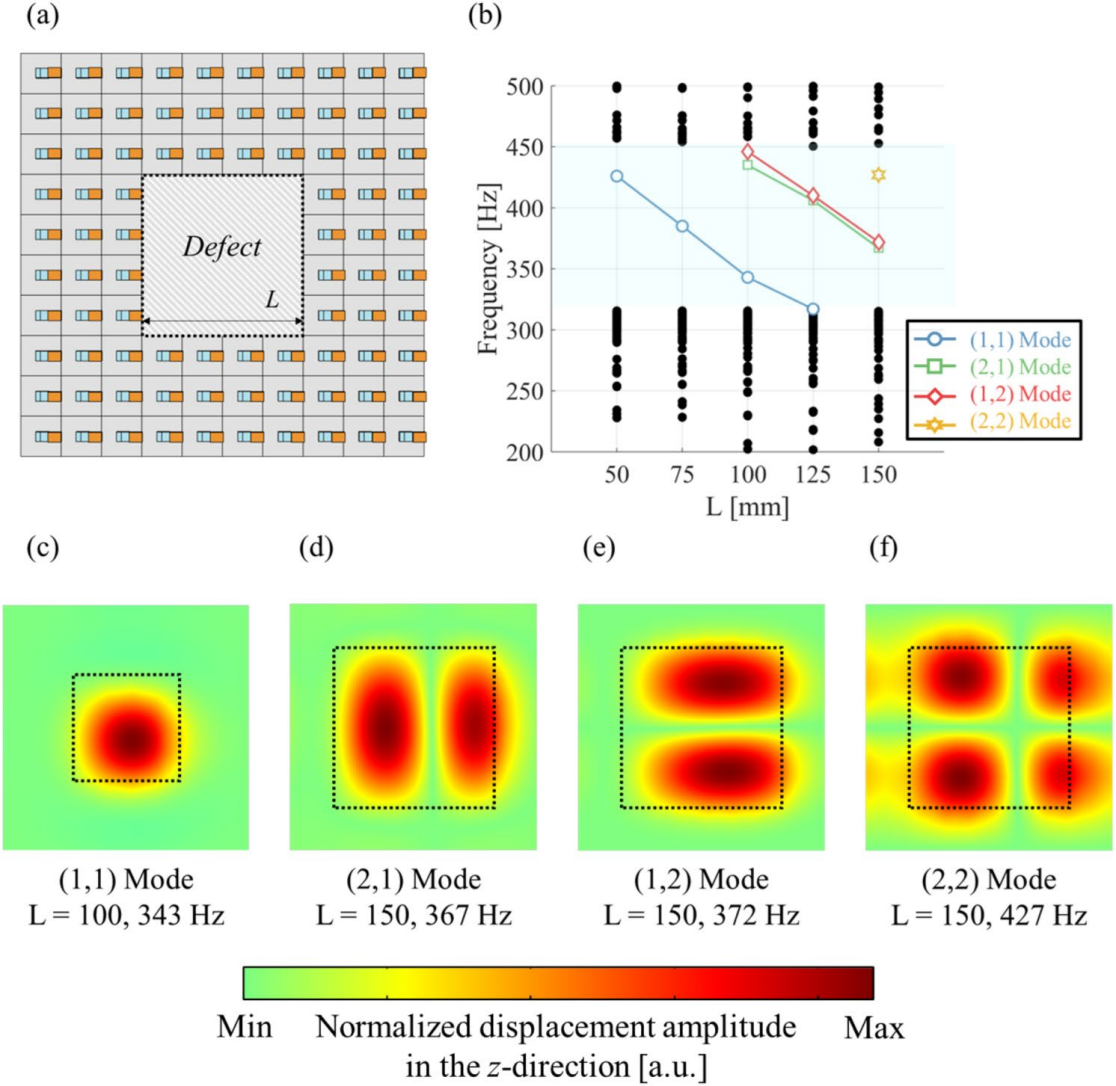
(**a**) Supercell comprising the a-LRM unit cells of a $\:10\times\:10$ array; a square defect of width *L* is formed at the center. (**b**) Change in defect modes as the width *L* of the defect increases. (**c**-**f**) Results for various defect-mode shapes

Defect-mode characteristics, such as the generation of mode frequencies and mode shapes, are significantly affected by the defect size. The defect itself exhibited the behavior of an effective plate in which the displacements were clamped at all edges. The effective bending stiffness of the defect-induced effective plate decreases as the defect size increases. Thus, in order to investigate the effect of the defect size on flexural defect modes, we perform a dispersion analysis for a supercell comprising the a-LRM unit cells of a 10 × 10 array with a square defect of width *L* at the center, as shown in Fig. 9a. It can be clearly seen that the generating frequency of the first-order defect mode (that is, the (1, 1) mode, Fig. 9c) gradually decreases, and the higher-order defect modes (that is, the (2, 1), (1, 2), and (2, 2) modes, Fig. 9d-f) newly arise in the defect band, as shown in Fig. 9b. However, once the defect size exceeded a specific threshold of *L* = 150 mm, the (1, 1) defect mode disappeared from the defect band. Thus, determining the appropriate defect size through the dispersion analysis of a supercell comprising a-LRM unit cells is important.

### Flexural vibration localization and amplification in a-LRM

**Fig. 10:**
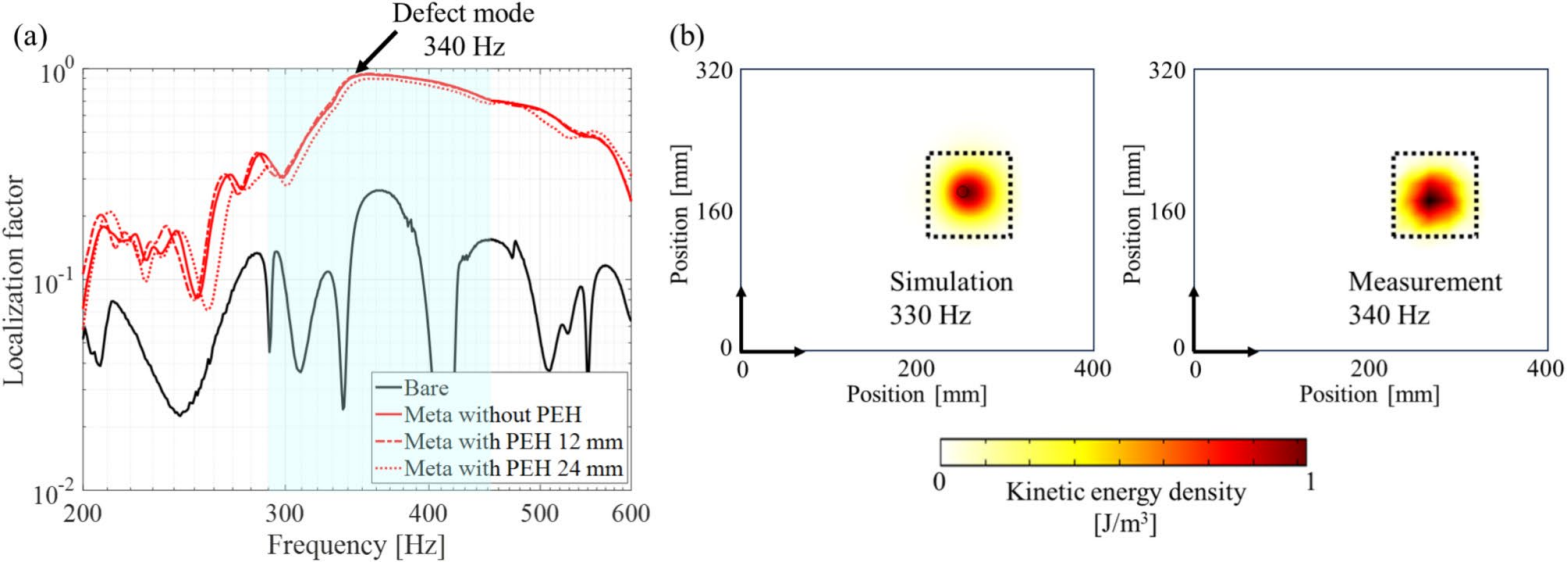
Characterization of flexural vibration localization and amplification in a-LRM. (**a**) Frequency response of measured localization factors for flexural waves. (**b**) Simulated and experimental flexural kinetic energy densities for the (1,1) defect mode

In Fig. 10a, the meta-plate shows a larger localization factor response over a broad frequency range below 1 kHz compared to the bare plate without a-LRMs (bare). Additionally, even when PEHs of different sizes (12 and 24 mm) were attached to the metaplate, there were slight changes in the localization factor responses. This suggests that the size and coupling effects of the PEH were negligible in the target frequency range of interest.

The overall behaviors were similar when comparing the frequency response of the localization factor obtained through the time-harmonic numerical analysis in Fig. 6b with that in Fig. 10a; only the defect mode frequencies between the two exhibit a slight difference of 10 Hz. This is thought to be because in the numerical model, it is difficult to accurately reflect (1) the actual bonding condition of the a-LRMs attached to the fabricated defect bandgap structure and (2) the clamped boundary condition of its exterior boundaries. Despite this difference, the flexural kinetic energy was still localized and amplified, as shown in Fig. 10b. Therefore, a defect bandgap structure with a-LRMs is highly suitable for low-frequency EH.

### Piezoelectric energy harvesting performance using the a-LRM

**Fig. 11:**
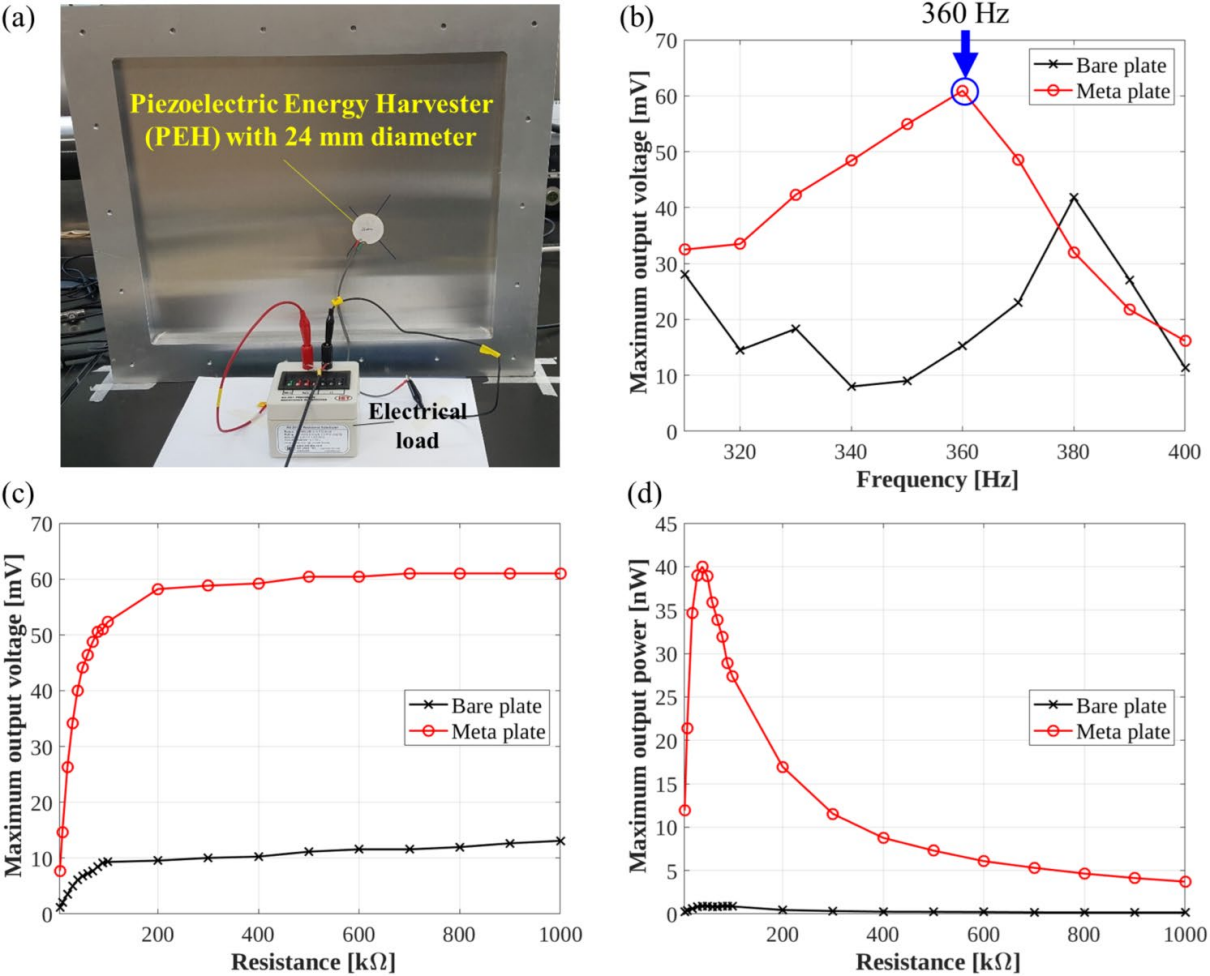
Piezoelectric EH performance in the defect bandgap structure (meta-plate) with a-LRMs (meta) and the bare plate (bare): For the bare plate, the PEH is installed at the same position as in the defect bandgap structure. (**a**) EH performance measurement setup configuration, where the PEH was attached to the front side of the defect bandgap structure. (**b**) Maximum output voltage response at various frequency conditions covering the bandgap measured at a fixed load resistance value of $\:1\:\text{M}{\Omega\:}$. (**c**) Maximum output voltage performance with respect to load resistances. (**d**) Maximum output power performance with respect to the load resistances

A circular piezoelectric ceramic disc $\:(23\text{P}\text{b}\text{Z}\text{r}{\text{O}}_{3}-36\text{P}\text{b}\text{T}\text{i}{\text{O}}_{3}-41\text{P}\text{b}\left(\text{N}\text{i}1/3\text{N}\text{b}2/3\right){\text{O}}_{3},{\text {23PZ-36PT-41PNN}})$ [27] with a diameter of 24 mm and a thickness of 2 mm was fabricated and used as the PEH, considering the energy localization area. The disc was attached at the center of the defect for the EH and connected to an electrical load so that the voltage and power across various electrical loading conditions could be measured, as shown in Fig. 11a. Additionally, for the bare plate without a-LRMs, the PEH was installed at the same position as that in the defect bandgap structure to act as a reference. To characterize the EH performance of the defect bandgap structure, we applied sinusoidal signals at each designated frequency within the range of 300–400 Hz. An oscilloscope was used as the data-acquisition system to measure the electrical output voltage generated by the PEH in the time domain. The PEH was connected to an electrical circuit with a simple resistive load so that the output performance, such as voltage and power, can be measured across various electrical impedance conditions.

Figure 11b–d present the EH performance results measured for the defect bandgap structure and bare plate without a-LRMs. The previous flexural energy localization and amplification experiment (Fig. 10) showed that the maximum amplification performance occurred near 340 Hz. We then measured the open-circuit voltage against the excitation frequency to identify the frequency point at which the harvesting performance was maximized. The maximum output voltage was measured by sweeping the excitation frequency with a step frequency of 10 Hz, while the load resistance was fixed at $\:1\:\text{M}{\Omega\:},$ which is sufficiently large to measure the open-circuit voltage.

Figure 11b shows the frequency response of the measured maximum output voltage. The maximum value of the output voltage is 60.9 mV in the defect bandgap structure, which is approximately 4.5 times of that from the bare plate (13.1 mV at the same excitation frequency. This confirmed the localization and amplification capabilities of the defect structures. The frequency at which the output voltage was maximized was 360 Hz, which was slightly different from the generating frequency of the (1, 1) defect mode (340 Hz) showed in Fig. 10. This frequency shift was due to the intrinsic electromechanical coupling effect of the PEH, which changed the mechanical resonance frequency of the PEH as the electrical load resistance increased. Moreover, the output voltage increased over a wide frequency range (approximately 300–380 Hz), including the target bandgap, demonstrating the broadband performance of the defect bandgap structure.

The measured maximum output voltages with respect to different electrical-load resistance levels, ranging from$\:\:0\:$to$\:\:1\:\text{M}{\Omega\:}\:$are illustrated in Fig. 11c. The output voltages increase and converge to the open-circuit output voltage of 60.9 mV in the defect bandgap structure and 13.1 mV in the bare plate without a-LRMs as the electrical-load resistance increases.

In Fig. 11d, the electrical output power is calculated and plotted using Ohm’s law ($\:{V}^{2}/R)$, where $\:V$ is the measured maximum output voltage in the time domain and $\:R$ is the simple load resistance across the circuit. With the a-LRM structure, an output power of 40.0 nW is achieved at an optimal load resistance level of $\:40\:\text{k}{\Omega\:}$ in the defect bandgap structure; this output power value is approximately 43-times of 0.92 nW, which is the output power value obtained from the bare plate. Hence, the power amplification ratio was approximately 43. These experimental results suggest that the flexural EH system based on our defect bandgap structure with a-LRMs can efficiently produce an amplified output power within a compact space while isolating the vibration energy within the targeted area and frequency.

**Fig. 12:**
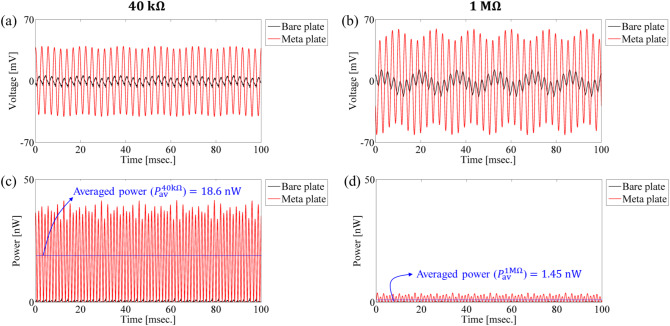
The experimentally measured time responses of the output voltage for (**a**) $\:40\:\text{k}{\Omega\:}$ and (**b**) $\:1\:\text{M}{\Omega\:}$, and the experimentally measured time responses of the output power for (**c**) $\:40\:\text{k}{\Omega\:}$ and (**d**) $\:1\:\text{M}{\Omega\:}$

Moreover, we applied a single-tone harmonic excitation of 360 Hz to the meta-plate and measured the time response of output voltage and output power over a duration of 100 ms for two load resistances: $\:40\:\text{k}{\Omega\:}$ and $\:1\:\text{M}{\Omega\:}$, so as to assess how the energy harvester actually responds at this frequency and to evaluate its energy conversion performance. First, as shown in Fig. 12a and b, the maximum output voltage is measured to be about 40 mV on average across the time range for the optimal load resistance of $\:40\:\text{k}{\Omega\:}$, while for the open-circuit condition of $\:1\:\text{M}{\Omega\:}$ load resistance, it was about 61 mV. When considering output power, which is crucial for evaluating PEH performance, the power generated at $\:40\:\text{k}{\Omega\:}$ was significantly higher than that at $\:1\:\text{M}{\Omega\:}$, as presented in Fig. 12c and d, highlighting the importance of the power-optimal resistance condition. Indeed, the mean power averaged over the time range $\:{P}_{\text{a}\text{v}}^{40\text{k}{\Omega\:}}$ for $\:40\:\text{k}{\Omega\:}$ is approximately 13-times higher than that $\:{P}_{\text{a}\text{v}}^{1\text{M}{\Omega\:}}$ for $\:1\:\text{M}{\Omega\:}$, i.e., $\:{P}_{\text{a}\text{v}}^{40\text{k}{\Omega\:}}/{P}_{\text{a}\text{v}}^{1\text{M}{\Omega\:}}\simeq\:12.8$. Furthermore, examining Fig. 12a and b, the time responses of the output voltage exhibit an envelope that includes a lower frequency component of approximately 60 Hz, which corresponds to the electrical frequency. This effect is particularly pronounced at the higher resistance of $\:1\:\text{M}{\Omega\:}$.

## Conclusions

In this study, we demonstrated a compact low-frequency flexural vibration EH and suppression system based on a defect-bandgap structure with a-LRMs. The a-LRM unit cell was fabricated using a simple acrylic cantilever beam and a titanium cylinder-shaped mass that is readily available in the market; thus, it can be easily mass-produced. Moreover, it does not damage the target object because it is detachable. The EH system was successfully realized with superior localization and amplification capabilities based on a supercell with a defect created by intentionally removing some a-LRMs, thereby maximizing the flexural vibration EH performance. The numerical and experimental results showed that the output power improved over a wide frequency range of approximately 310–380 Hz, including the generating frequency of the (1,1) defect mode. Particularly, the harvesting power via the defect mode was significantly enhanced by a factor of 43 compared with that of the bare plate without a-LRMs. It is worthy to note that tuning the bandgap has very significant impact on the energy harvesting performance and vibration isolation performance. The frequency response of the Purcell factor to characterize the defect or cavity itself showed that the vibration energy is dramatically enhanced by the defect modes at 340 Hz and 430 Hz by the bandgap structure. Since the proposed defect bandgap structure exploited these modes to achieve energy harvesting and vibration isolation in this study, if one wants to tailor those performances to a specified target, tuning bandgap can be considered as the first way. Also, although existing metamaterial/PC-based EH studies assumed that waves were incident on the system from outside, we considered that the input wave location was specified inside the system. The proposed low-frequency vibration EH system can be used as an effective EH in various applications where the wave input location can be specified, including industrial, defense, and home appliances.

## Data Availability

The datasets used and/or analyzed during the current study are available from the corresponding author on reasonable request.

## Abbreviations

PC: Phononic crystal
EH: Energy harvesting
GRIN: Gradient-index
PEH: Piezoelectric energy harvester
HR: Helmholtz resonator
PVDF: Polyvinylidene difluoride
LRM: Locally resonant metamaterial
a-LRM: Attachable locally resonant metamaterial
FE: Finite element
